# Toxicity responses of Cu and Cd: the involvement of miRNAs and the transcription factor SPL7

**DOI:** 10.1186/s12870-016-0830-4

**Published:** 2016-06-28

**Authors:** Heidi Gielen, Tony Remans, Jaco Vangronsveld, Ann Cuypers

**Affiliations:** Environmental Biology, Centre for Environmental Sciences, Hasselt University, Agoralaan Building D, Diepenbeek, B-3590 Belgium

**Keywords:** Copper (Cu), Cadmium (Cd), miRNA, SPL7, Arabidopsis, Metal stress, Cu deficiency, Cu homeostasis

## Abstract

**Background:**

MicroRNAs are important posttranscriptional regulators of gene expression playing a role in developmental processes as well as in stress responses, including metal stress responses. Despite the identification of several metal-responsive miRNAs, the regulation and the role of these miRNAs and their targets remain to be explored. In this study, miRNAs involved in the response to Cd and Cu excess in *Arabidopsis thaliana* are identified. In addition, the involvement of the transcription factor SPL7, namely the key regulator of Cu homeostasis, in these metal stress responses is demonstrated by the use of an *spl7* knockout mutant. Furthermore, more insight is given in the Cd-induced Cu deficiency response through determining the effects of adding supplemental Cu to Cd-exposed plants.

**Results:**

Thirteen miRNAs were identified in response to Cu and Cd excess in *A. thaliana*. Several of these miRNAs (miR397a, miR398b/c and miR857) were oppositely affected under Cu and Cd exposure. The induced expression of these miRNAs after Cd exposure was totally abolished in the *spl7* mutant (SQUAMOSA promoter binding protein like7), indicating a major role for SPL7 in the Cd response. Plants exposed to Cd showed a higher Cu content in the roots, whereas the Cu content in the leaves of the *spl7* mutant was reduced. Furthermore, the Cd-induced Cu deficiency response disappeared when supplemental Cu was added.

**Conclusions:**

Copper- and Cd-responsive miRNAs were identified and several of them are SPL7-dependently regulated. SPL7 seems to be a shared component between both the Cu toxicity and the Cd toxicity response, yet oppositely regulated, that is inactivated after Cu exposure and activated after Cd exposure. Since SPL7 is the key regulator of Cu homeostasis, and Cd affects the Cu homeostasis, we hypothesize that SPL7 is activated in response to Cd possibly due to a Cd-induced Cu deficiency. Since adding additional Cu to Cd-exposed plants resulted in the disappearance of the Cu deficiency response, Cd possibly provokes Cu deficiency, thereby activating SPL7 and inducing subsequently the Cu deficiency response.

**Electronic supplementary material:**

The online version of this article (doi:10.1186/s12870-016-0830-4) contains supplementary material, which is available to authorized users.

## Background

Plants require mechanisms to control growth, development and stress responses and therefore an accurate regulation of gene expression is essential. In recent years, the recognition of microRNAs (miRNAs) as important posttranscriptional regulators of gene expression has emerged. MiRNAs are non-protein coding small endogenous RNAs of approximately 21 nucleotides that are complementary with target mRNA thereby causing its translational repression or cleavage [1].

Over the last decade, cloning and sequencing of small RNAs and computational analysis have revealed many miRNAs. Most of these annotated miRNAs have a role in the regulation of developmental processes, like organ morphogenesis and polarity, meristem boundary, floral patterning, vascular development, lateral root development and stomatal development [2–6]. However, different functional studies also showed the participation of miRNAs in stress responses. Liu *et al* [7] identified 14 miRNAs induced by high-salinity, drought and low temperature in *Arabidopsis thaliana* on a microarray-based analysis, among which miR168, miR171 and miR396 responded to all three stresses. MiR395, negatively regulating ATP sulfurylases and a sulphate transporter, was strongly induced upon sulphate starvation [8–10]. In addition, phosphate starvation induced the expression of miR399, which targets an E2 conjugase PHOSPHATE2, that functions upstream of a subset of phosphate starvation-induced genes [11–13]. Additionally, Pant *et al.* [14] identified also other P- and N-starvation responsive miRNAs in *A. thaliana*. These results demonstrate that miRNAs are affected by a fluctuating nutrient availability and are essential in regulating nutrient homeostasis.

Besides this, miRNAs are also involved in responses to metal stress in diverse plant species [15]. Zhou *et al.* [16] identified six miRNAs responsive to Cd, mercury (Hg) and aluminium (Al) in *Medicago truncatula*. Recent studies also reported altered expression of some miRNAs in *Brassica napus* [17, 18] and in rice [19, 20] after Cd exposure. It should be noted however that different metals can have different responses upon miRNA expression. On the one hand, the expression of miR398 is downregulated by copper (Cu) and iron (Fe), causing increased copper/zinc superoxide dismutase (CSD) expression [21, 22]. On the other hand, excess cadmium (Cd) induces miR398 and this response is also seen under low Cu availability [22, 23]. The induction of the miR398 expression under Cu deprivation is regulated by the active transcription factor SQUAMOSA promoter binding protein-like7 (SPL7) assumed to be a central regulator in Cu homeostasis [24].

Despite these examples, information about other miRNAs involved in the response to Cd and Cu excess in *A. thaliana* is still rather scarce. In this study, we used an RT-qPCR based pri-miRNA platform [14] to identify Cd- and Cu-responsive (pri-)miRNAs. The expression of their target genes was measured to unravel the role of these miRNAs in the Cd and Cu stress response. In addition, the involvement of the transcription factor SPL7 in these metal stress responses was demonstrated by the use of an *spl7* knockout mutant and more insight was gained in the Cd-induced Cu deficiency response through determining the impact of adding supplemental Cu to Cd-exposed plants.

## Results

### Plant fresh weight is reduced after exposure to Cu and Cd

**Fig. 1:**
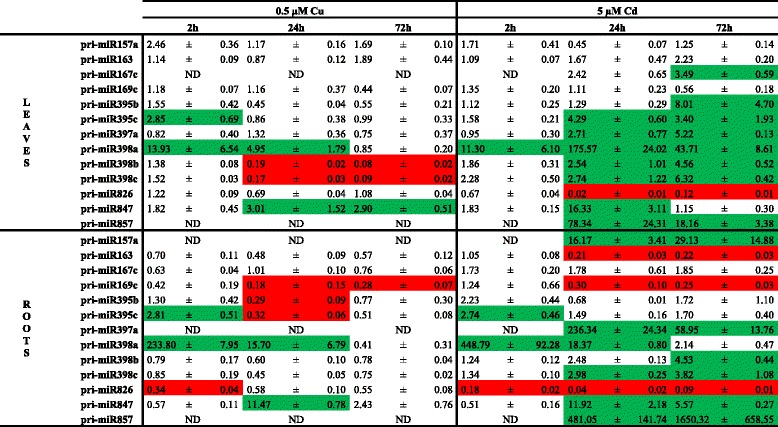
Fresh weight in roots and leaves of *A. thaliana* plants. Nineteen-days-old plants were exposed for 2, 24 or 72 h to 5 μM CdSO_4_, 0.5 μM CuSO_4_ or grown under control conditions. Data are mean ± S.E. of 8 biological replicates. Significant differences (*P* < 0.05) after non-parametrical ANOVA test (Kruskal-Wallis) and correction with pairwise Wilcoxon rank sum test are indicated with different capital (roots) or small (leaves) letters

Root and leaf fresh weight (FW) of wildtype (WT) plants were determined in control conditions and after exposure to 0.5 μM Cu and 5 μM Cd for 0, 2, 24 and 72 h (Fig. 1). The FW of the roots and leaves increased over time in all conditions. However, after 72 h exposure to Cd and Cu root FW was significantly lower compared to controls, while leaf FW was not.

### Exposure to excess Cu and Cd induces lipid peroxidation

**Fig. 2:**
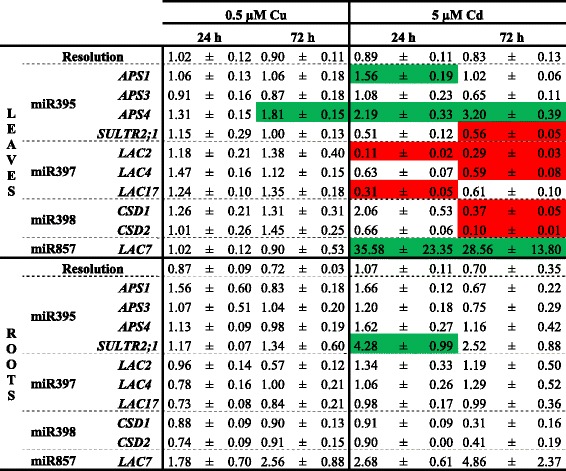
Lipid peroxidation in roots and leaves of *A. thaliana* plants. Nineteen-days-old plants were exposed for 2, 24 or 72 h to 5 μM CdSO_4_, 0.5 μM CuSO_4_ or grown under control conditions. Data are mean ± S.E. of at least 3 biological replicates. Significant differences (*P* < 0.05) after two-way ANOVA test and Tukey correction are indicated with different small letters (roots)

Since excess metals are considered to affect plasma membranes as a primary target, lipid peroxidation in the roots and leaves of Cu- and Cd-exposed plants was determined (Fig. 2). In the roots, a significant increase in lipid peroxidation was found in Cu-exposed plants already after 2 h of exposure, while in the Cd-exposed roots, there was only a significant increase in lipid peroxidation from 24 h onwards. Lipid peroxidation was significantly higher in 0.5 μM Cu-exposed roots than in the roots after 5 μM Cd exposure, but for both metals the increase in lipid peroxidation did not increase further over time. No changes in lipid peroxidation were detected in the leaves after 0.5 μM Cu and 5 μM Cd exposure.

### Several miRNAs are Cd and/or Cu responsive

To determine the involvement of miRNAs in Cd and Cu stress responses in *A. thaliana*, we screened the pri-miRNA expression levels of 180 miRNAs (Additional file 1: Table S1) using an RT-qPCR platform [14]. Based on a threshold (at least 2.5 fold change in normalized expression), thirteen Cd and/or Cu responsive pri-miRNAs were identified in roots and leaves. In the roots, six pri-miRNAs were differentially expressed after 0.5 μM Cu exposure and eleven after exposure to 5 μM Cd (Table 1). In the leaves, five pri-miRNAs exhibited a different expression after exposure to 0.5 μM Cu and ten pri-miRNAs after 5 μM Cd exposure (Table 1).

**Table 1:**
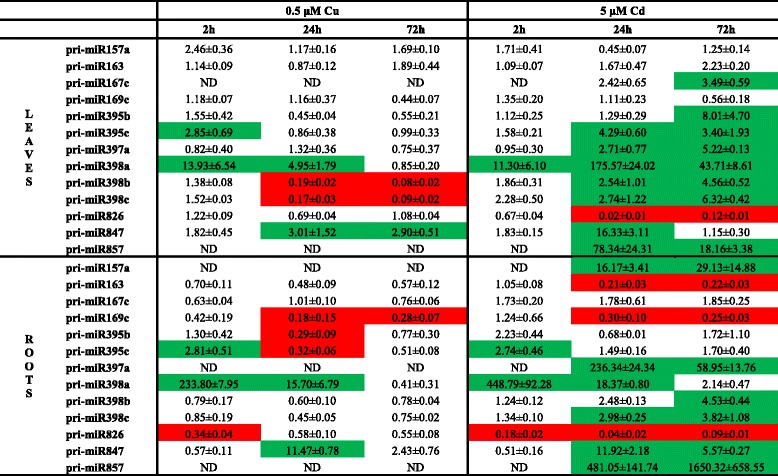
Gene expression levels of pri-miRNAs in roots and leaves of *A. thaliana* plants. Nineteen-days-old plants were exposed for 2, 24 or 72 h to 0.5 μM CuSO_4_, 5 μM CdSO_4_ or grown under control conditions. Only the pri-miRNAs with an expression level that is 2.5 times up (green) or down (red) regulated are shown. Transcript levels were calculated relative to the non-exposed plants at each time point. Data are mean ± S.E. of 3 biological replicates. (ND, not detected after 40 cycli)

Metal exposure changed the expression of miRNAs already after short exposure time. Three miRNAs had an altered expression at the earliest time-point of 2 h: in roots and leaves an upregulation of pri-miR395c after Cu exposure and of pri-miR398a after Cd and Cu exposure was observed, while a downregulation of pri-miR826 in Cu- and Cd-exposed roots was noticed. However, most of the responsive pri-miRNAs were induced or repressed after 24 h and 72 h of exposure (Table 1). A time-dependent strengthening of the effect only seems to be the case for the induction of pri-miR857 expression in the roots of 5 μM Cd-exposed plants on one hand and for the reduction of pri-miR398b and pri-miR398c expression in the leaves after 0.5 μM Cu exposure on the other hand (Table 1). The induced expression of pri-miR398a in the Cd- and Cu-exposed roots and in the Cu-exposed leaves diminished over time and even disappeared after 72 h (Table 1).

Some pri-miRNAs showed an opposite expression pattern after exposure to Cu or Cd. The expressions in the leaves of pri-miR398b and c were repressed after 24 h and 72 h of Cu exposure and induced after 24 h and 72 h of Cd exposure (Table 1). Moreover, several pri-miRNAs seemed to be Cd-specific in this experimental design, by which is meant that these pri-miRNAs (in roots: pri-miR157a, pri-miR397a and pri-miR857; in leaves: pri-miR167c and pri-miR857) were only expressed after exposure to Cd and not (or very lowly) expressed in control conditions as well as after Cu exposure (Table 1).

### Target gene expression levels of Cd- and Cu-responsive miRNAs

Since miRNAs negatively regulate their target mRNA, identification of these targets provides knowledge about the pathways where these miRNAs are involved in. To determine if the Cd- and/or Cu-induced differences in pri-miRNA expressions were translated into altered target expression levels under these conditions, a gene expression analysis of several of these targets (validated by [9, 23, 25]) was performed after 24 h and 72 h exposure to 0.5 μM Cu or 5 μM Cd (Table 2). There were almost no alterations in target gene expressions in plants exposed to 0.5 μM Cu. Only in the leaves after 72 h of Cu exposure, the slightly reduced expression of pri-miR395b (Table 1) was translated into a significant upregulation of its target ATP sulfurylase 4 (*APS4)* (Table 2). Also in the roots of 5 μM Cd-exposed plants the only transcript level induced after 24 h exposure was from sulphate transporter 2;1 (*SULTR2;1)*, a target of miR395 (Table 2). Nevertheless, in Cd-exposed leaves, multiple changes in expression levels of target genes were observed. An induced expression of the miR395 targets *APS1* and *APS4,* and the miR857 target laccase 7 (*LAC7)* was found in Cd-exposed leaves, but these altered target expressions were not in accordance with the expression profiles of the miRNAs. Alternatively, the expression levels of *LAC2, LAC4* and *LAC17* decreased in the leaves after Cd exposure, while the expression of the regulating miR397 increased. Similarly, the gene expression levels of the miR395 target *SULTR2;1* and the miR398 targets *CSD1* and *CSD2* decreased in the leaves after 72 h Cd exposure (Table 2), while the expression of their regulating miRNAs increased (Table 1).

**Table 2:**
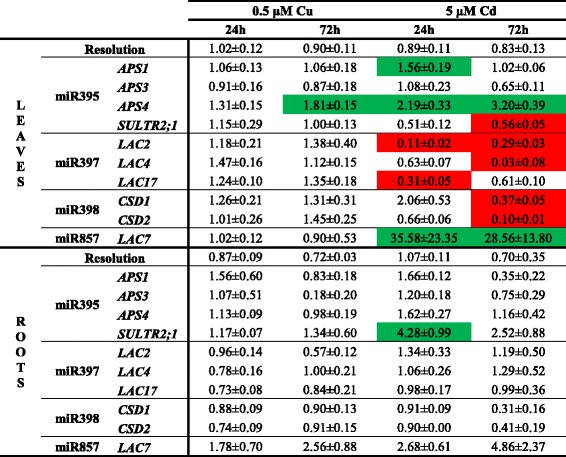
Gene expression levels of pri-miRNA targets in roots and leaves of *A. thaliana* plants. Nineteen-days-old plants were exposed for 24 or 72 h to 0.5 μM CuSO_4_, 5 μM CdSO_4_ or grown under control conditions. Transcript levels were calculated relative to the non-exposed plants at each time point. Data are mean ± S.E. of 3 biological replicates. Significant differences (*P* < 0.05) relative to the non-exposed plants at each time point after one-way ANOVA test and Tukey correction are indicated in color (upregulation, green; downregulation, red)

### The involvement of the transcription factor SPL7 in the Cd and Cu response

The transcription factor SPL7, already known to be important for Cu homeostasis in plants [24], binds to GTAC motifs in the promoters of several miRNAs (cupro-miRNAs) thereby upregulating their expression [24]. A number of these cupro-miRNAs, namely miR397a, miR398b, miR398c and miR857 [24], were also Cd responsive (Table 1). Therefore, Cu and Cd responses were further investigated using an *spl7* knockout mutant to define a possible role for SPL7 in these responses.

#### Copper and Cd sensitivity of root growth in wild-type plants and the spl7 mutant

**Fig. 3:**
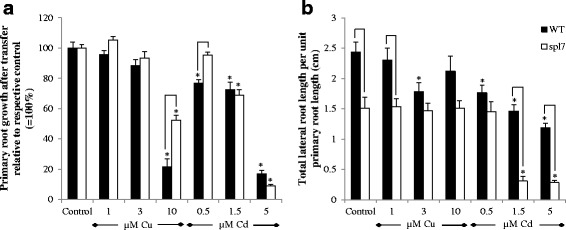
Root growth parameters of *A. thaliana* wildtype and *spl7* knockout plants. Primary root growth after transfer **a** is expressed relative to the control for each genotype and total lateral root length **b** is expressed per unit primary root length. Seven-days-old plants were exposed for another 7 days to 1, 3 and 10 μM CuSO_4_ or 0.5, 1.5 and 5 μM CdSO_4_ or further grown under control conditions. Data are given as the mean ± S.E. of at least 11 biological independent replicates. Significant differences (*P* < 0.05) after two-way ANOVA test (primary root growth) and Tukey correction or after non-parametrical ANOVA test (Kruskal-Wallis; lateral root length) and correction with pairwise Wilcoxon rank sum test are indicated as following: treatment difference with an asterisk (*) and genotype difference with connection lines

Sensitivity of root growth of wild-type and *spl7* mutants to excess Cd and Cu was tested in vertical agar plates. After germination on control plates, homogenous seedlings with a primary root length of approximately 2.5 cm and 2 cm for WT and *spl7* mutant respectively were transferred to plates containing a concentration range of Cd or Cu. The growth rate of the primary root was equal between both genotypes after transfer (data not shown). Therefore, the primary root growth after transfer was significantly lower in the *spl7* mutant compared to WT plants under control conditions (2.48 ± 0.05 cm and 3.04 ± 0.12 cm respectively), and also the total lateral root length per unit primary root length was smaller in the mutant (Fig. 3). Exposure to 10 μM Cu significantly inhibited primary root length in both genotypes, but this inhibition was less pronounced in the *spl7* mutants (Fig. 3a). On the other hand, total lateral root length per unit primary root length was lower after 3 μM Cu exposure in WT plants, whereas there was no effect in the *spl7* mutant (Fig. 3b). After transfer to Cd-containing plates, primary root growth and total lateral root length per unit primary root length were already significantly inhibited at 0.5 μM Cd in WT plants, while in the *spl7* mutant inhibition was observed from 1.5 μM Cd exposure onwards (Fig. 3). Total lateral root length per unit primary root length, however, was significantly more inhibited at higher concentrations (1.5 and 5 μM Cd) in the *spl7* mutant compared to WT plants (Fig. 3b).

#### Effect of Cd and excess Cu on fresh weight, lipid peroxidation and metal content

**Fig. 4:**
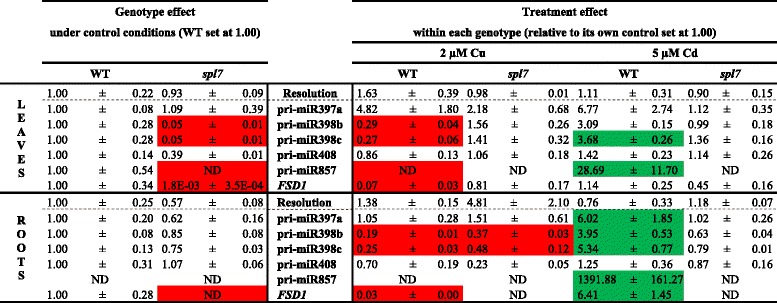
Fresh weight in leaves and roots of *A. thaliana* wildtype and *spl7* knockout plants. Nineteen-days-old plants were exposed for 24 h or 72 h to 2 μM CuSO_4_, 5 μM CdSO_4_ or grown under control conditions. Data of the leaves (upper bars) and roots (lower bars) are given as the mean ± S.E. of 5 biological independent replicates. Significant differences (*P* < 0.05) after three-way ANOVA test and Tukey correction are indicated as following: treatment difference with an asterisk (*), time difference with a tilde (~) and genotype difference with connection lines

In the following experiments, Cu concentrations were raised to 2 μM Cu since the *spl7* mutant appeared to be less sensitive to Cu than WT plants. The effect of 2 μM Cu and 5 μM Cd after 24 h and 72 h of exposure on fresh weight (FW) of both roots and leaves was determined (Fig. 4). After 24 h exposure, there was a significantly decreased root FW after 2 μM Cu exposure in both genotypes. After 72 h exposure, the FW of roots and leaves in both genotypes was reduced after Cu and Cd exposure compared to control plants, except for Cd-exposed *spl7* mutant roots. Whereas leaf FW of both genotypes increased over time in all conditions, this was also the case for root growth of the *spl7* mutant, but not for Cu- and Cd-exposed roots of WT plants, leading to a significant genotype difference in root FW after 72 h Cu exposure (Fig. 4).

Whereas a significant increase in lipid peroxidation after 24 h exposure to 2 μM Cu was observed in both leaves and roots, 5 μM Cd exposure had no effect on lipid peroxidation. Under all conditions, there was no genotype difference in lipid peroxidation (Additional file 2: Figure S1).

**Fig. 5:**
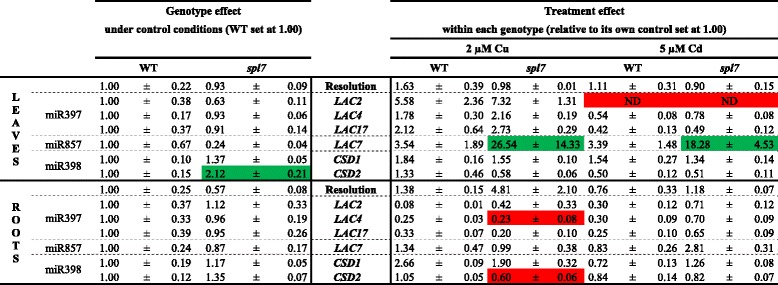
The translocation factor of copper and cadmium from root to leaves. Nineteen-days-old *A. thaliana* wildtype and *spl7* knockout plants were exposed for 24 or 72 h to 2 μM CuSO_4_, 5 μM CdSO_4_ or grown under control conditions. The translocation factor (TF) of Cu (**a**) and Cd (**b**) were calculated as the percentage of the concentration in the leaves relative to the concentration in the roots. Data are given as the mean ± S.E. of 5 biological independent replicates. Significant differences (*P* < 0.05) after non-parametrical ANOVA test (Kruskal-Wallis) and correction with pairwise Wilcoxon rank sum test are indicated as following: treatment difference with an asterisk (*) and genotype difference with connection lines

The Cu and Cd content in roots and leaves of both genotypes after 24 h and 72 h exposure to 2 μM Cu and 5 μM Cd were determined (Table 3). In roots and leaves of WT and *spl7* mutant plants, Cu or Cd concentrations significantly increased after exposure to 2 μM Cu or 5 μM Cd respectively (Table 3). However, there were genotype differences in root metal content. In roots, *spl7* mutant plants had a significantly lower Cu content as compared to WT plants after 24 h Cu exposure, while their root Cd contents were significantly higher after 72 h Cd exposure (Table 3). Although the Cu content increased in the roots of both genotypes after 72 h Cd exposure in comparison with the control plants, the leaf Cu content was reduced in *spl7* mutant plants and not in WT plants at this time point (Table 3). Alterations in metal uptake under control and exposed conditions also led to differences in the metal translocation factor (TF). The strong increase in root Cu content after 24 h and 72 h Cu exposure led to a significantly reduced Cu TF under both conditions (Fig. 5a). Interestingly, also Cd exposure significantly reduced the Cu TF in both WT and *spl7* mutant plants. Furthermore, the genotype difference in root Cd content after 72 h Cd exposure also led to a genotype difference in the Cd TF that was significantly lower in the *spl7* mutant (Fig. 5b).

**Table 3:** Copper and cadmium content in A. thaliana wildtype and spl7 knockout plants

			24 h		72 h	
			WT	*spl7*	WT	*spl7*
Cu	leaves	Control	7.50 ± 0.53^abc^	6.53 ± 0.37^bc^	8.95 ± 0.81^abe^	9.76 ± 0.66^ade^
		2 μM Cu	12.43 ± 0.32^d^	11.63 ± 0.44^de^	16.70 ± 1.33^f^	16.79 ± 0.74^f^
		5 μM Cd	6.31 ± 0.21^bc^	4.93 ± 0.74^c^	6.82 ± 0.17^abc^	4.64 ± 0.48^c^
	roots	Control	25.00 ± 2.81^abc^	25.61 ± 1.93^abc^	18.91 ± 1.18^c^	23.47 ± 2.24^bc^
		2 μM Cu	1069.45 ± 30.28^d^	712.47 ± 38.00^e^	943.04 ± 43.13^de^	854.71 ± 50.80^de^
		5 μM Cd	29.97 ± 2.50^ab^	27.38 ± 2.18^ab^	45.85 ± 1.71^f^	34.45 ± 2.04^af^
Cd	leaves	5 μM Cd	514.45 ± 6.05^a^	699.38 ± 156.61^a^	1831.70 ± 53.69^b^	1549.40 ± 151.09^b^
	roots	5 μM Cd	884.79 ± 116.14^a^	1282.86 ± 152.62^a^	2421.08 ± 163.42^b^	3034.21 ± 113.16^c^

Nineteen-days-old plants were exposed for 24 or 72 h to 2 μM CuSO4, 5 μM CdSO4 or grown under control conditions. Copper and Cd content (mg kg-1 DW-1) in roots and leaves were calculated. Data are given as the mean ± S.E. of 5 biological independent replicates. Significant differences (P < 0.05) after three-way ANOVA test (Cu content) or two-way ANOVA test (Cd content; time/genotype) and Tukey correction are indicated with different small letters (per metal, per organ)

#### Gene expression levels of SPL7-regulated Cd- and Cu-responsive (pri-)miRNAs and their targets

The role of SPL7 in miRNA expression related to target gene expression during Cd and Cu stress was further explored with a gene expression analysis. Transcript levels of five pri-miRNAs (pri-miR397a, pri-miR398b, pri-miR398c, pri-miR408 and pri-miR857) and one gene (iron superoxide dismutase1, *FSD1*), all with GTAC motifs in their promoters, were determined in roots and leaves of WT and *spl7* mutant plants exposed during 24 h to 2 μM Cu or 5 μM Cd (Table 4). There was a clear genotype difference between WT and the *spl7* mutant plants under control conditions. In the roots, the transcript level of *FSD1* was reduced (not detectable) in the *spl7* mutant compared to WT plants, while in the leaves the expression of pri-miR398b, pri-miR398c, pri-miR857 and *FSD1* was significantly lower in the *spl7* mutant (Table 4). For the WT genotype, similar Cu and Cd treatment differences were obtained as in the screening experiment (Table 1). In the roots, exposure to 2 μM Cu for 24 h significantly reduced the expression level of pri-miR398b and pri-miR398c in both genotypes and of *FSD1* in WT plants. In the leaves, Cu exposure significantly lowered transcript levels of pri-miR398b, pri-miR398c, pri-miR857 and *FSD1* in WT plants, while the low expression levels of the pri-miRNAs in the *spl7* mutant under control conditions remained (Table 4). On the other hand, after 24 h exposure to 5 μM Cd, there was a significantly upregulated expression in WT roots of *FSD1* and all pri-miRNAs, except pri-miR408, and an upregulated expression in WT leaves of pri-miR398c and pri-miR857. These responses were absent in Cd-exposed *spl7* mutants (Table 4).

**Table 4:**
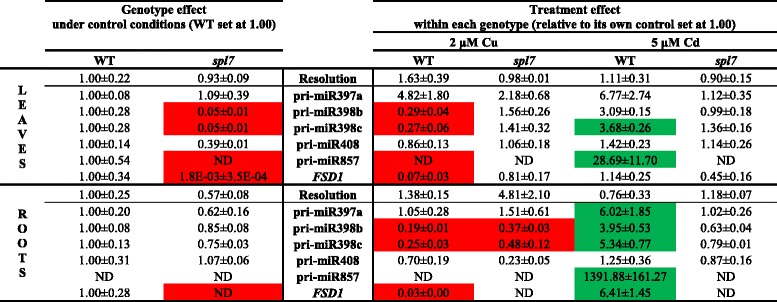
Gene expression levels of pri-miRNAs in *A. thaliana* wildtype and *spl7* knockout plants. Nineteen-days-old plants were exposed for 24 h to 2 μM CuSO_4_, 5 μM CdSO_4_ or grown under control conditions. Data of the genotype effect of the *spl7* mutant are given as the fold changes ± S.E. of at least 3 biological independent replicates relative to WT plants (set at 1.00). Data of the treatment effect within each genotype are mean ± S.E. of at least 3 biological replicates relative to its own control (set at 1.00). Significant differences (*P* < 0.05) after one-way (genotype effect) or two-way (treatment effect) ANOVA test and Tukey correction are indicated in color (upregulation, green; downregulation, red). (ND, not detected after 40 cycli)

The effects of Cd and Cu on the expression levels of the miRNA-regulated targets are given in Table 5 and are described per miRNA. Neither in roots nor in leaves of both genotypes significant changes were found in the expression levels of miR397 targets *LAC2*, *LAC4* and *LAC17* under control conditions and after Cu exposure, except for a decreased transcript level of *LAC4* in the roots after Cu exposure. In addition, *LAC2* expression in the leaves was not detectable anymore after 5 μM Cd exposure in both genotypes (Table 5). Investigating the expression level of the miR857 target *LAC7* revealed that neither a genotype nor a treatment effect was observed in the roots. In the leaves however, Cu exposure significantly increased the expression of *LAC7* in the *spl7* mutant. Exposure to Cd significantly led to an upregulation of the pri-miR857 expression level in the leaves of the WT plants while this remained undetectable in the *spl7* mutant (Table 4). However no significant changes were found for *LAC7* expression in WT plants, whereas a significant induction was observed in *spl7* mutants (Table 5). Concerning the expression levels of the miR398 targets *CSD1* and *CSD2*, no genotype effect was noticed in the roots under control conditions (Table 5). Although Cu exposure reduced the expression of pri-miR398b and c in both genotypes in the roots (Table 4), there was only a significant reduction present in the expression of *CSD2* in the *spl7* mutant (Table 5). There were no significant Cd treatment effects on the *CSD* transcript levels in the roots, which corresponds with the unaltered pri-miR398b and c expression levels in the *spl7* mutant, but not with the Cd-induced expression of pri-miR398b and c in the WT roots. In the leaves, under control conditions the significantly lower transcript levels of pri-miR398b and c of the *spl7* mutant compared to WT plants (Table 4) led to an increased expression of *CSD2* in the *spl7* mutant (Table 5). Furthermore, although in the WT leaves the expression of pri-miR398b and c was significantly downregulated after Cu exposure and upregulated after Cd exposure (only pri-miR398c) and in the *spl7* mutant the expression of pri-miR398b and c remained low after Cu and Cd exposure, there were no significant Cu and Cd treatment effects in the leaves on the expression of the *CSDs*.

**Table 5:**
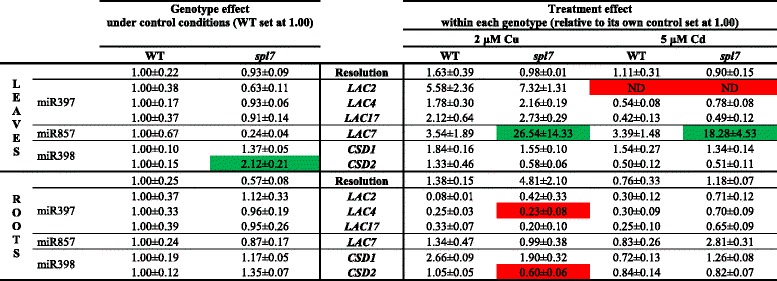
Expression levels of targets of SPL7 regulated miRNAs in *A. thaliana* wildtype and *spl7* knockout plants. Nineteen-days-old plants were exposed for 24 h to 2 μM CuSO_4_, 5 μM CdSO_4_ or grown under control conditions. Data of the genotype effect of the *spl7* mutant are given as the fold changes ± S.E. of at least 3 biological independent replicates relative to WT plants (set at 1.00). Data of the treatment effect (within each genotype) are mean ± S.E. of at least 3 biological replicates relative to its own control (set at 1.00). Significant differences (*P* < 0.05) after one-way (genotype effect) or two-way (treatment effect) ANOVA test and Tukey correction are indicated in color (upregulation, green; downregulation, red). (ND, not detected after 40 cycli)

### The impact of adding additional Cu to Cd-exposed plants

Since SPL7 is involved in the Cd response and Cu homeostasis is disturbed after Cd exposure, knowing the impact of adjustments to external Cu concentrations in combination with Cd exposure will provide more insights in the Cd-induced Cu deficiency responses. Therefore, WT plants were simultaneously exposed to 5 μM Cd and increasing concentrations of Cu (0.5, 1 or 2 μM Cu additionally added to the Hoagland solution) for 72 h whereafter metal content and gene expression analyses were performed.

#### Additional Cu restores Cu levels and reduces Cd levels in leaves of Cd-exposed plants

**Fig. 6:**
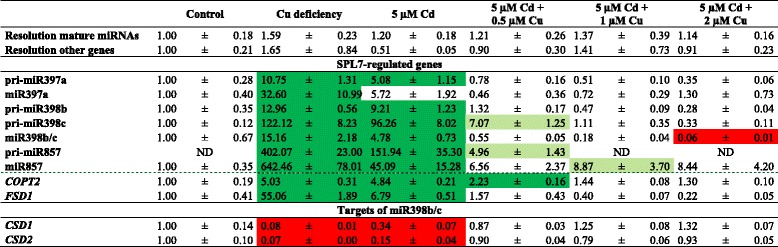
Copper and Cd content in leaves and roots of wildtype plants. Nineteen-days-old plants were further grown under control conditions or Cu deficiency, or were exposed to 5 μM Cd and supplemented with extra Cu (0, 0.5, 1 or 2 μM Cu extra compared to control Hoagland solution) for 72 h. Copper and Cd contents were calculated in mg per kg dry weight (mg kg^-1^ DW^-1^). Data are mean ± S.E. of 6 biological replicates. Significant differences (*P* < 0.05) after one-way ANOVA test and Tukey correction are indicated with different letters

Exposure of plants to 5 μM Cd resulted in decreased Cu levels in the leaves, while after exposure to Cd in combination with extra Cu (1 and 2 μM Cu) there was no difference in leaf Cu content compared to control plants (Fig. 6a). In the roots, Cu levels were increased after exposure to 5 μM Cd and adding extra Cu to Cd-exposed plants increased Cu levels even more (Fig. 6c). On the contrary, the addition of extra Cu significantly reduced Cd content in leaves and roots compared to exposure to Cd only (Fig. 6b and d).

#### The Cd-induced Cu deficiency response disappears with supplemental Cu

**Fig. 7:**
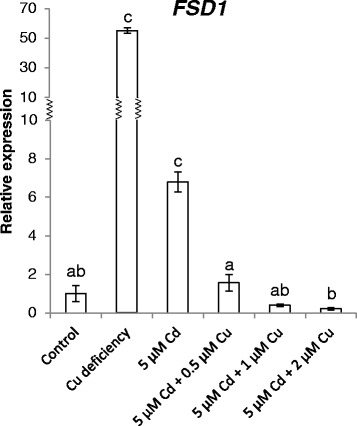
Transcript levels of *FSD1* in leaves of wildtype plants. Nineteen-days-old plants were further grown under control conditions or Cu deficiency, or were exposed to 5 μM Cd and supplemented with extra Cu (0, 0.5, 1 or 2 μM Cu extra compared to control Hoagland solution) for 72 h. Transcript levels were calculated relative to the non-exposed plants (controls). Data are mean ± S.E. of 5 biological replicates. Significant differences (*P* < 0.05) after one-way ANOVA test and Tukey correction are indicated with different letters

The effect of adding supplemental Cu to Cd-exposed plants was further explored with a gene expression analysis in the leaves. The transcript level of *FSD1* was induced in the leaves of plants grown under Cu deficiency or after exposure to 5 μM Cd. However, this induction disappeared in plants exposed to 5 μM Cd in combination with extra Cu (0.5, 1 and 2 μM) (Fig. 7). In general, the same response was observed for the transcript levels of all other measured SPL7-regulated pri-miRNAs, mature miRNAs (miR397a, miR398b/c, miR857) and the gene *COPT2* (Table 6). Whereas the increased expression after Cd exposure did not always completely disappear when extra Cu was added, most transcript levels significantly decreased. Furthermore, the expression of *CSD1* and *CSD2* reduced in Cu deficient and Cd-exposed plants, while in the combined application of Cd and supplemental Cu, there was no difference in *CSD1* and *CSD2* transcript levels as compared to control plants (Table 6).

**Table 6:**
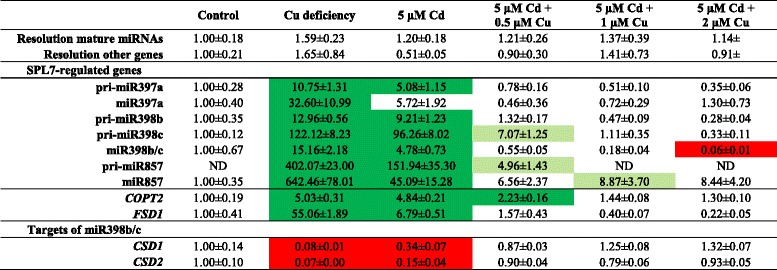
Gene expression levels of SPL7-regulated genes and targets of miR398b/c in the leaves of *A. thaliana.* Nineteen-days-old plants were exposed for 72 h to 5 μM CdSO_4_ with 0, 0.5, 1 or 2 μM CuSO_4_ extra, or grown under Cu deficiency from germination on or under control conditions. Transcript levels were calculated relative to the non-exposed plants. Data are mean ± S.E. of at least 5 biological replicates. Significant differences (*P* < 0.05) after one-way ANOVA test and Tukey correction are indicated with colour shading: red for reduction compared to control, dark green for induction compared to control and light green for induction compared to control but reduction compared to 5 μM Cd

## Discussion

Plant growth and development is challenged due to the occurrence of several environmental stressors, including elevated metal concentrations. Therefore, an appropriate regulation of stress responses is of major importance. MiRNAs have a regulatory role in numerous stresses, including metal stress [15]. High-throughput expression profiling of plants exposed to metals have identified several metal-responsive miRNAs [19, 20, 26–30], however the regulation of these miRNAs and the precise role of these miRNAs and their targets in metal responses remain to be explored. Therefore, identifying Cu- and Cd-responsive miRNAs in *A. thaliana* and their regulation after Cu and Cd exposure represent important research questions.

To identify Cu- and Cd-responsive miRNAs in *A. thaliana*, we exposed 19-days-old plants for 2, 24 and 72 h to 0.5 μM Cu, 5 μM Cd or grew them under control conditions. The applied metal concentrations in our study are sublethal and based on the concentrations found in pore water of sandy soils in polluted areas of Belgium [31–33]. Whether the applied metal concentrations were toxic was verified at the morphological level [impaired root development (Fig. 1)] and at the cellular level [induced lipid peroxidation (Fig. 2)]. These parameters were previously shown to be affected upon Cd and Cu exposure [22, 34–39].

In order to clarify whether Cu- and Cd-induced responses are dependent on miRNAs, we analyzed the expression profile of 180 pri-miRNAs in Cu- and Cd-exposed roots and leaves. Pant *et al.* [14] validated the use of a pri-miRNA profiling platform as a useful screening tool. Although it is recently demonstrated that the level of pri-miRNAs not always reflects the level of its mature miRNA [40], other studies have shown the correlation between pri-miRNA and mature miRNA levels [11, 14] as was also observed for the majority of the analysed miRNAs in our study (Table 6). Until lately, miR398 was the only miRNA family that was known to be involved in Cu and Cd stress in *A. thaliana* [21, 22]. But recently Barciszewska-Pacak *et al.* [40] identified more than 40 *Arabidopsis* pri-miRNAs responsive to (among others) Cd and Cu excess using an RT-qPCR mirEX platform. The Cd and Cu responsive pri-miRNAs that we identified in our experimental setup (Table 1), showed no altered expression in their study, except for pri-miR398a/b/c after Cu exposure, which could be due to the different experimental setups. Barciszewska-Pacak *et al.* [40] screened the pri-miRNA expression in leaves of plants grown on 0.8 % agar 1/2MS plates exposed for 24 h to 10 μM Cu and 10 μM Cd [40], while we used both leaf and root samples of plants grown in hydroponic culture exposed during 2, 24 and 72 h. Although fast alterations in miRNA expression levels after 0.5 μM Cu and 5 μM Cd were observed in our study, a negative correlation with the transcript levels of their targets was not always seen, especially not in the Cu-exposed plants (Table 2). However, Cd exposure resulted in the leaves in induced expression levels of miR395, miR397 and miR398 and this led to reduced transcript levels of their targets *SULTR2;1*, *LAC2, LAC4* and *LAC17,* and *CSD1* and *CSD2* (Tables 1 and 2). Cuypers *et al.* [22] already demonstrated an upregulation of miR398b together with a downregulation of *CSD1* and *CSD2* in *A. thaliana* after 24 h exposure to 5 μM Cd. The negative correlations between Cd-responsive miRNAs and their targets show the importance of miRNAs in regulatory networks.

In this study, we exposed plants to Cu and Cd, two metals that have different physico-chemical properties, i.e. Cu is an essential nutrient and redox-active whereas Cd is nonessential and not redox-active. Striking is the opposite regulation after Cu and Cd exposure of miR398b/c in the leaves and the specific Cd-responsive miRNAs, namely miR157a, miR167c, miR397a and miR857 that were not (or very low) expressed under control conditions or after Cu exposure (Table 1). Some of these miRNAs, more specifically miR397a, miR398b/c and miR857 all have several GTAC motifs in their promoter regions, which are binding places for the transcription factor SQUAMOSA promoter binding protein-like7 (SPL7) [24]. SPL7 is assumed to be the key regulator of Cu homeostasis. Under Cu deficiency, it activates transcription of the so-called cupro-miRNAs (miR397, miR398b/c, miR408 and miR857) targeting Cu-containing proteins, i.e. laccases and Cu/Zn superoxide dismutases [24]. Additionally, GTAC motifs are present in the promoters of several Cu-transporters, such as *COPT1*, *COPT2* and *COPT6*, and a copper chaperone *CCH* and the expression of all these genes is increased under Cu deficiency through the action of SPL7 [41]. Because of the SPL7-dependent decrease of non-essential or replaceable Cu-containing proteins and the SPL7-dependent increase of Cu transporters under Cu deficiency, it is suggested that the limited available Cu is preferred for plastocyanin in photosynthesis and Cu uptake in the roots is stimulated [24]. In general, after Cu excess the expression of the cupro-miRNAs (miR397a, miR398b/c and miR857) were decreased or not detectable at all (Table 1). In addition, exposure to Cd increased the transcript levels of these cupro-miRNAs (Table 1). The induced expression of miR398b/c after Cd exposure was already demonstrated in previous studies [22, 41], but this regulation was not yet seen for miR397a and miR857 in *A. thaliana* (Table 1).

Gayomba *et al.* [41] demonstrated that SPL7 is involved in the Cd-induced increase of miR398, as well as in the upregulated expression of *FSD1* and *COPT1/2/6*. Therefore, we wanted to confirm this SPL7 dependency of the Cd-induced Cu deficiency response in our experimental setup with expansion of the data to all cupro-miRNAs and their targets. Furthermore, we were also interested in the possible involvement of SPL7 in the response to Cu excess. To analyse this, we used an *spl7* knockout mutant. The Cu and Cd sensitivity of the *spl7* mutant was determined based on root growth of plants grown on vertical agar plates and exposed to a range of Cu and Cd concentrations. On one hand, primary root growth (Fig. 3a), lateral root length per unit primary root length (Fig. 3b) and root FW (Fig. 4) was less or not diminished in Cu-exposed *spl7* mutants compared to WT plants. On the other hand, exposure to Cd reduced primary root growth and lateral root length (Fig. 3) in both genotypes, however, lateral root length per unit primary root length decreased tremendously more in the *spl7* mutant. Therefore we can conclude that the *spl7* mutant is less sensitive to excess Cu and more sensitive to Cd toxicity compared to the WT plants.

**Fig. 8:**
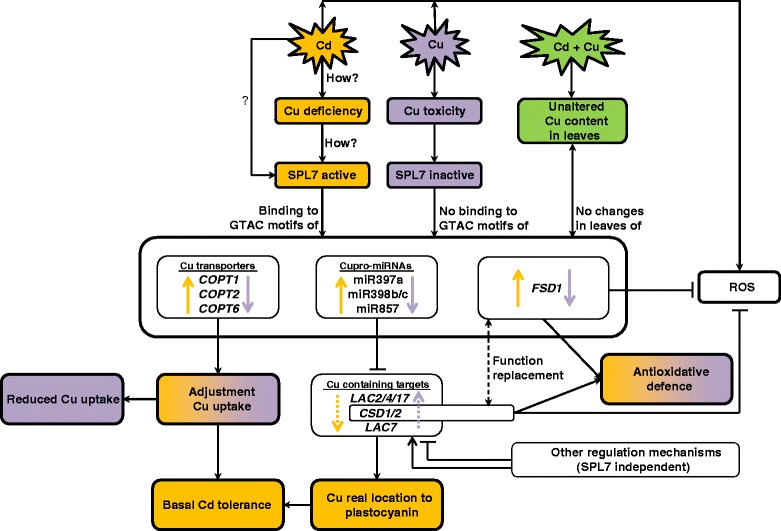
Schematic model for the (in)activation of SPL7 and the subsequent regulations under Cd and Cu exposure. Cd exposure (orange colored) elicits a Cu deficiency response through the activation of SPL7. Active SPL7 binds to GTAC motifs in the promoters of several Cu transporters, cupro-miRNAs and other genes like *FSD1* thereby inducing their expression and decreasing miRNA target transcription. This Cu deficiency response, resulting in an increased Cu uptake and reallocation of Cu to plastocyanin, is required for basal Cd tolerance. The Cd-induced *FSD1* expression counterbalances the decrease in *CSD1/2* transcript levels to maintain the antioxidative defence against superoxide radicals. Conversely, Cu toxicity (purple colored) inactivates SPL7 resulting in decreased transcription levels of all these SPL7-regulated genes. The regulations of all cupro-miRNA targets are not always clear (indicated by the dotted arrows) since they are also subjected to other SPL7-independent regulation mechanisms. Interestingly, when Cd exposure is combined with extra Cu (green colored), Cu homeostasis remains unaltered in the leaves, resulting in neither Cd-induced Cu deficiency responses nor Cu toxicity responses in the leaves

We wanted to confirm and expand our knowledge about the involvement of SPL7 in Cd toxicity stress responses and to determine whether SPL7 also plays a role in Cu toxicity stress responses. Therefore, gene expression levels of cupro-pri-miRNAs and their targets were measured in hydroponically grown WT and *spl7* mutant plants after exposure to 2 μM Cu and 5 μM Cd for 24 h. Since the *spl7* mutant is less sensitive to Cu than WT plants, Cu concentrations were elevated to 2 μM Cu, which is still a sublethal environmentally realistic concentration. Although SPL7 has been put forward as the major regulator of Cu deficiency and a central regulator of Cu homeostasis [24], SPL7 also plays a role under control conditions since the total knockout of SPL7 in the *spl7* mutant led to a downregulation of several miRNAs (pri-miR398b and c, pri-miR408 and pri-miR857) (Table 4). This is conform with the findings of Gayomba *et al.* [41] where transcript levels of other genes with a GTAC-containing promoter (*COPT2*, *COPT6*, *FSD1* and *miR398*) were reduced in the *spl7* mutant. We found that exposure to excess Cu in general downregulates the cupro-miRNAs in WT plants and that the already very low expression of these cupro-miRNAs in the *spl7* mutant remained low after excess Cu (Table 4, Fig. 8). Previous studies also demonstrated the downregulation of miR398 after Cu exposure in *A. thaliana* [21, 22]. This response to Cu toxicity is comprehensible since the opposite is expected compared to Cu deficiency, with no activation of SPL7 and hence, no induction of transcription of these miRNAs and genes (Fig. 8). In contrast, while Cu toxicity generally resulted in a downregulation of these cupro-miRNAs, Cd toxicity induced the transcript levels of miR397a, miR398b/c, miR857 and *FSD1* (Table 4, Fig. 8). However, this induced expression of cupro-miRNAs and *FSD1* in WT plants after Cd exposure was totally abolished in the *spl7* mutant (Table 4) just like it is demonstrated for the expression of *COPT1/2/6* [41]. With these findings we confirm that the Cd-induced Cu deficiency response depends on SPL7 [41] (Fig. 8).

In order to verify whether the targets of the SPL7-regulated miRNAs are also under the control of SPL7 after Cu and Cd exposure, target gene expression levels were measured. Clear up- or downregulation of the cupro-miRNAs (Table 4) is not always leading to alterations in target expression levels (Table 5), since the observed target gene transcript levels are a result of both, transcriptional activity regulated by other transcription factors and the SPL7-dependent miRNA-mediated post-transcriptional breakdown (Fig. 8). Multiple studies describe the reduced expression of miR398b/c and induction of *CSD* transcript levels after exposure to excess Cu [21, 22]. This is in accordance with our findings where a downregulation of miR398b/c and a maintained or small upregulation of *CSDs* were seen (Tables 4 and 5). In addition, it has been shown that excess Cu leads to an elevated production of reactive oxygen species (ROS) to which plants have to defend themselves [22, 34, 42]. Therefore, the putatively SPL7-dependent miR398-mediated maintenance of *CSD* transcript levels can be a first-line defence against Cu-induced oxidative stress. Nevertheless, Cd also induces oxidative stress ([43] and references therein), but compared to the Cu toxicity response, *CSD* transcript levels were decreased in our study (Table 2). However, alternatively Cd induced *FSD1* expression (Table 4), also regulated by SPL7, to maintain the SOD capacity (Fig. 8).

It is known that Cd competes with other essential elements for cellular uptake sites and for binding sites in metalloenzymes, thereby disturbing the homeostasis of these elements [42, 44]. For example, Yoshihara *et al.* [45] and Xu *et al.* [46] demonstrated the disturbance of the iron homeostasis after Cd exposure at morphological and molecular level. In this regard, Cd possibly also interferes with Cu homeostasis (Fig. 8) as shown by the higher Cu content in the roots after exposure to Cd (Table 3 and Fig. 6c) and by the induction of several Cu transporters (Table 6 and Fig. 8) [41]. This disturbance in Cu homeostasis, causing Cu deficiency, possibly can result in the activation of SPL7, the major regulator for Cu homeostasis in *A. thaliana*. This subsequently results into upregulations of the Cu transporters and cupro-miRNAs and hence downregulations of their targets as seen in this study (Tables 4,5,6 and Fig. 8). In addition, Gayomba *et al.* [41] stated that this SPL7-dependent Cu deficiency response, i.e. Cu uptake and reallocation, is required for basal Cd tolerance in *A. thaliana*.

However, how SPL7 is activated and how Cd induces the Cu deficiency response is still unknown. Recently, a working model for the regulation of SPL7 function was reported [47] as well as several scenarios for the link between Cd and SPL7 activation proposed [41]. On one hand it is proposed that Cd could directly interact with SPL7 or on the other hand could alter the cellular Cu availability and hence affect SPL7 activation [41]. Therefore, in a subsequent experiment, extra Cu was supplemented (0.5, 1 and 2 μM Cu) to Cd-exposed plants and the Cd-induced Cu deficiency responses were analysed. The decreased Cu content in leaves of Cd-exposed plants (Fig. 6a) [41] restored to control level when extra Cu was added, while the opposite was seen for Cd (Fig. 6b). In addition, at the transcript level, the observed Cu deficiency response after Cd exposure disappeared when extra Cu was added (Table 6). Taken together, these data support our hypothesis that adding extra Cu to Cd-exposed plants restores Cu levels in leaves resulting in the disappearance of the Cu deficiency response. Thus, Cd possibly provokes Cu deficiency, thereby activating SPL7 and inducing subsequently the Cu deficiency response (Fig. 8). However, whether the disappearance of the Cd-induced Cu deficiency response after adding extra Cu is due to restored Cu levels or due to reduced Cd levels should be further investigated.

## Conclusions

In this study, we identified several miRNAs that are involved in responses to Cu and Cd excess. Copper and Cd are physico-chemically different metals and have an opposite effect on the expression of some Cu transporters, miRNAs (miR397a, miR398b/c, miR857) and the gene *FSD1* (Fig. 8), that all have GTAC binding sites for the transcription factor SPL7 in their promoter. SPL7 seems to be a shared component between both the Cu toxicity and the Cd toxicity response, yet oppositely regulated, that is inactivated after Cu exposure and activated after Cd exposure (Fig. 8). Since SPL7 is the key regulator of Cu homeostasis, and Cd affects the Cu content in the roots and the Cu translocation to the shoots, we hypothesize that the involvement of SPL7 in the Cd response is possibly due to a Cd-induced Cu deficiency (Fig. 8). How Cd induces Cu deficiency and how SPL7 is activated remain interesting research topics that require further investigation.

## Methods

### Plant culture, exposure and harvest

The *spl7* knockout mutant *A. thaliana* line (Col-0 background; SALK_125385C; Alonso *et al.* [48]) was obtained from the European Arabidopsis Stock Centre (uNASC) and was confirmed by PCR to be a homozygous T-DNA insertion line (Additional file 3: Figure S2).

Wild-type (WT) and mutant seeds were surface sterilized and grown in hydroponic culture [49] in the same conditions as described by Keunen *et al.* [35]. After 19 days, Hoagland solution was supplemented with 0.5 μM CuSO_4_, 2 μM CuSO_4_, 5 μM CdSO_4_ or 5 μM CdSO_4_ + extra CuSO_4_ (0.5, 1 or 2 μM CuSO_4_) and plants were harvested after an exposure time of 2, 24 and 72 h, to examine immediate responses, responses after 1 full circadian day and responses at metabolic homeostasis respectively. Copper deficient plants were grown from germination on in adapted Cu deficient Hoagland solution (0.5 nM Cu). Root and leaf (entire rosette) samples were taken, snap frozen in liquid nitrogen and stored at -70 °C prior to lipid peroxidation analysis and gene expression analysis. Samples for element analysis were dried prior to extraction.

For root growth analysis, seeds were surface sterilized and grown on vertical agar plates. The preparation of the germination plates was performed as described by Remans *et al.* [38] with an adjusted Cu concentration (final concentration of 100 nM) and after 7 days of growth, seedlings were transferred to treatment plates and exposed for another 7 days to 1, 3 and 10 μM CuSO_4_ or 0.5, 1.5 and 5 μM CdSO_4_. Plates were scanned on a flatbed scanner at 600 dpi and root growth was analysed using the Optimas6 Image Analysis Software (Media Cybernetics).

### Element analysis

Fresh root samples (175–400 mg FW) were washed for 15 min with 10 mM Pb(NO_3_)_2_ at 4 °C and rinsed with distilled water, while leaf samples (400–1000 mg FW) were only rinsed with distilled water. Samples were oven-dried (60 °C for 3 weeks), weighed, digested with 70 % HNO_3_ in a heat block and dissolved in 5 ml of 2 % HCl. Copper and Cd concentrations were measured via inductively coupled plasma-atomic emission spectrometry (ICP-AES, Perkin-Elmer, 1100B, USA). Blanks (only HNO_3_) and standard references (NIST Spinach 1570a) were included.

### Lipid peroxidation analysis

The amount of thiobarbituric acid reactive metabolites (TBArm), as a measure of lipid peroxidation, was determined spectrophotometrically. Roots and leaves (50–100 mg FW) were homogenized in 1 ml of 0.1 % trichloroacetic acid (TCA), centrifuged (10 min, 20000 g, 4 °C) and 3.5 times diluted in 0.5 % TBA. After heating to 95 °C for 30 min, samples were put on ice for 5 min and centrifuged. The absorbance was measured at 532 nm and corrected for unspecific absorbance at 600 nm. The amount of TBA reactive metabolites was calculated using the law of Beer-Lambert (ε = 155 mM^-1^ cm^-1^).

### Gene expression analysis

Frozen tissues (50–75 mg FW) were disrupted using two stainless steel beads and the Retsch Mixer Mill MM2000 (Retsch, Haan, Germany) under frozen conditions. Total RNA was extracted using the RNAqueous Kit (Life Technologies, Carlsbad, CA, USA) according to manufacturer’s instructions. RNA concentration and purity was determined spectrophotometrically on the NanoDrop ND-1000 (ThermoScientific, Wilmington, DE, USA). The cDNA for the pri-miRNA screening experiment had an RNA input of 3.2 μg that was DNase treated (Turbo DNA-freeTM kit, Life Technologies) and reverse transcribed using SuperScriptTM III Reverse Transcriptase (Life Technologies) in a 30 μl reaction according to manufacturer’s instructions. The cDNA for the experiment using the *spl7* knockout mutant, has an RNA input of 1 μg that was DNase treated (Turbo DNA-freeTM kit, Life Technologies) and reverse transcribed using the High-Capacity cDNA Reverse Transcription Kit (Life Technologies) according to manufacturer’s instructions. A 10-fold dilution of all the cDNA of both experiments was made using 1/10 diluted TE buffer (1 mM Tris-HCL, 0.1 mM EDTA, pH 8.0, Sigma-Aldrich, Belgium) and stored at -20 °C.

Quantitative PCR analysis was performed with the 7900HT Fast Real-Time PCR System (Life Technologies). For the pri-miRNA screening, the pri-miRNA platform [14] was used and reactions contained Fast SYBR Green Master Mix (Life Technologies), 500 nM of a gene-specific forward and reverse primer, and 1 μl of the diluted cDNA in a final volume of 10 μl. For target genes, primers were designed using primer express 2.0 and a BLAST was performed (http://www.arabidopsis.org/Blast/index.jsp) to check specificity (Additional file 4: Table S2). A two-fold dilution series of a pooled sample (all cDNAs of the experiment) was used to create a standard curve for the evaluation of primer efficiencies that were accepted when they were within a range between 80 and 100 % (measured over 6 dilution points). PCR amplifications were performed at universal cycling conditions and contained Fast SYBR Green Master Mix, 300 nm of a gene-specific forward and reverse primer, and 2.5 μl of the diluted cDNA in a final volume of 10 μl.

For the gene expression analysis of mature miRNAs, total RNA was extracted using the mirVana miRNA isolation kit (Life Technologies, Carlsbad, CA, USA) according to manufacturer’s instructions. RNA concentration and purity was determined spectrophotometrically on the NanoDrop ND-1000. Multiplex reverse transcription and Real-time qPCR were performed using Taqman microRNA assays (Life Technologies, Carlsbad, CA, USA).

All relative expression levels were calculated as 2^-ΔCq^ and normalized by the geometric average of 2^-ΔCq^ values of minimum three reference genes selected by the GrayNorm algorithm [50]. Data of treatment effects are expressed relative to the control of its own genotype set at 1.00. For some genes (pri-miR167c, pri-miR397a, pri-miR857) expression was not detected after 40 cycli, neither after 60 cycli under control conditions. In that case, cyclic threshold was set at 40 for the control samples and thereby treatment effects could be calculated and expressed relative to the control. The calculated and represented treatment effects are therefore minimal fold changes when expression was undetermined after 40 cycli under control conditions. All details of the workflow according to the Minimum Information for publication of Quantitative real-time PCR Experiments (MIQE) guidelines as described by Bustin *et al.* [51] are shown in Additional file 5: Table S3.

### Statistical analysis

Statistical analysis was performed using R (version 2.15.1; R Foundation for Statistical Computing, Vienna, Austria). Normal distribution of the datasets was tested using the Shapiro-Wilk test and homoscedasticity was evaluated with the Bartlett’s test. If necessary, transformations of the datasets were applied. Gene expression data were always log transformed. Significant differences were determined using ANOVA test and Tukey correction. If the assumption of normality was not fulfilled, a non-parametrical ANOVA test (Kruskal-Wallis) and correction with pairwise Wilcoxon rank sum test was applied.

## Additional files

Additional file 1: Cq values of 180 pri-miRNAs in leaves and roots measured using an RT-qPCR platform. (XLSX 124 kb) Additional file 2: Lipid peroxidation in leaves and roots of *A. thaliana* wild-type and *spl7* knockout plants. Nineteen-days-old plants were exposed for 24 h to 2 μM CuSO4, 5 μM CdSO4 or grown under control conditions. Data are mean ± S.E. of 4 biological replicates. Significant treatment differences (*P* < 0.01) after two-way ANOVA test and Tukey correction are indicated with an asterisk (*). There were no genotype differences. (DOCX 34 kb) Additional file 3: Verification of the T-DNA insertion in the *spl7* mutant. Primers were designed using T-DNA primer design. (DOCX 32 kb) Additional file 4: Forward and reverse primers used to determine gene expression levels via quantitative real-time PCR. E-E-jn, exon-exon junction; E-I-b, exon-intron boundary; YLS, yellow-leaf-specific; UBQ10, ubiquitin; ACT, actin ; EF, elongation factor; APS, ATP sulfurylase; SULTR, sulfate transporter; LAC, laccase; CSD, Cu/Zn superoxide dismutase; FSD, Fe superoxide dismutase. *Pri-miRNA primer concentrations were increased to 900 nM. (DOCX 18 kb) Additional file 5: Quantitative real-time PCR parameters according to the Minimum Information for publication of Quantitative real-time PCR Experiments (MIQE) guidelines derived from Bustin *et al.* [51]. (DOCX 16 kb)
