# Supplementary material for: Toxicity responses of Cu and Cd: the involvement of miRNAs and the transcription factor SPL7
Source: BMC Plant Biol. 2016 Jun 28;16:145. doi: 10.1186/s12870-016-0830-4 (PMC4924269; doi:10.1186/s12870-016-0830-4)
Supplement: Additional file 2: — Lipid peroxidation in leaves and roots of A. thaliana wild-type and spl7 knockout plants. Nineteen-days-old plants were exposed for 24 h to 2 μM CuSO4, 5 μM CdSO4 or grown under control conditions. Data are mean ± S.E. of 4 biological replicates. Significant treatment differences (P < 0.01) after two-way ANOVA test and Tukey correction are indicated with an asterisk (*). There were no genotype differences. (DOCX 34 kb) [file 12870_2016_830_MOESM2_ESM.docx]

**Additional file 2.** **Lipid peroxidation in leaves (A) and roots (B) of *A. thaliana* wildtype (black) and *spl7* knockout (white) plants**. Nineteen-days-old plants were exposed for 24 h to 2 µM CuSO_4_, 5 µM CdSO_4_ or grown under control conditions. Data are mean ± S.E. of 4 biological replicates. Significant treatment differences (P<0.01) after two-way ANOVA test and Tukey correction are indicated with an asterisk (*). There were no genotype differences.
