# Supplementary material for: Toxicity responses of Cu and Cd: the involvement of miRNAs and the transcription factor SPL7
Source: BMC Plant Biol. 2016 Jun 28;16:145. doi: 10.1186/s12870-016-0830-4 (PMC4924269; doi:10.1186/s12870-016-0830-4)
Supplement: Additional file 3: — Verification of the T-DNA insertion in the spl7 mutant. Primers were designed using T-DNA primer design. (DOCX 32 kb) [file 12870_2016_830_MOESM3_ESM.docx]

**Additional file 3. Verification of the T-DNA insertion in the *spl7* mutant**. Primers were designed using T-DNA primer design.
