# Supplementary material for: Toxicity responses of Cu and Cd: the involvement of miRNAs and the transcription factor SPL7
Source: BMC Plant Biol. 2016 Jun 28;16:145. doi: 10.1186/s12870-016-0830-4 (PMC4924269; doi:10.1186/s12870-016-0830-4)
Supplement: Additional file 4: — Forward and reverse primers used to determine gene expression levels via quantitative real-time PCR. E-E-jn, exon-exon junction; E-I-b, exon-intron boundary; YLS, yellow-leaf-specific; UBQ10, ubiquitin; ACT, actin ; EF, elongation factor; APS, ATP sulfurylase; SULTR, sulfate transporter; LAC, laccase; CSD, Cu/Zn superoxide dismutase; FSD, Fe superoxide dismutase. *Pri-miRNA primer concentrations were increased to 900 nM. (DOCX 18 kb) [file 12870_2016_830_MOESM4_ESM.docx]

**Additional file 4.** **Forward and reverse primers used to determine gene expression levels via quantitative real-time PCR**. E-E-jn, exon-exon junction; E-I-b, exon-intron boundary; YLS, yellow-leaf-specific; UBQ10, ubiquitin; ACT, actin ; EF, elongation factor; APS, ATP sulfurylase; SULTR, sulfate transporter; LAC, laccase; CSD, Cu/Zn superoxide dismutase; FSD, Fe superoxide dismutase. *Pri-miRNA primer concentrations were increased to 900 nM.

| **Locus** | **Annotation** | **Forward primer (5’-3’)** | **Reverse primer (5’-3’)** | **Exon location** | **Amplicon length (bp)** |
| --- | --- | --- | --- | --- | --- |
| **Reference genes** | | | | | |
| *AT2G28390* | *SAND family* | AACTCTATGCAGCATTTGATCCACT | TGATTGCATATCTTTATCGCCATC | Exon 13 and 14 | 61 |
| *AT5G15710* | *F-box protein* | TTTCGGCTGAGAGGTTCGAGT | GATTCCAAGACGTAAAGCAGATCAA | Exon 1 | 63 |
| *AT5G08290* | *YLS8* | TTACTGTTTCGGTTGTTCTCCATTT | CACTGAATCATGTTCGAAGCAAGT | 3’-UTR | 61 |
| *AT4G05320* | *UBQ10* | GGCCTTGTATAATCCCTGATGAATAAG | AAAGAGATAACAGGAACGGAAACATAGT | 3’-UTR | 61 |
| *AT3G18780* | *ACT2* | CTTGCACCAAGCAGCATGAA | CCGATCCAGACACTGTACTTCCTT | Exon 2 | 68 |
| *AT5G60390* | *EF1a* | TGAGCACGCTCTTCTTGCTTTCA | GGTGGTGGCATCCATCTTGTTACA | Exon 1 and E1-E2-jn | 76 |
| **Genes of interest** | | | | | |
| *AT3G22890* | *APS1* | AATTCCGATGGTAACCCCGT | TCGTACCCCATGTTCTAGCTATCCT | Exon 1 and exon 2 | 91 |
| *AT4G14680* | *APS3* | CTTGTTCATTTCTGGCACTAAGATGAG | AGGACTTTCCAGCCTCCAGG | E4-E5-jn and exon 5 | 93 |
| *AT5G43780* | *APS4* | GATTCCAACCAAGTCACACTCGT | CGTTCTTCTTTGGGATGCTTGTAA | Exon 2 and exon 3 | 91 |
| *AT5G10180* | *SULTR2;1* | CTTCCGTTGAGATCGGACTTCTT | CCAAGCGTTTCTATCCCTGGAC | Exon 6 and exon 7 | 91 |
| *AT2G29130* | *LAC2* | AGGAATCACGCGGCACTACC | TTTTCCGTTGACCGTCACG | Exon 1 and exon 2 | 91 |
| *AT2G38080* | *LAC4* | CTTCTCTGTGTTCCCAGCTCCA | GCTTGAGCATAATCTAGTCACGTTCTTC | Exon 1 and exon 2 | 91 |
| *AT3G09220* | *LAC7* | TGCGCTCTAATTCTTCTCGCA | GCCTAGACACCGTTAAGTTTTGTACGT | Exon 1 and E1-E2-jn | 91 |
| *AT5G60020* | *LAC17* | CTTCCTCAACCTGCATTTGGG | CAAGGCTCTTTGTGTGGCAAAG | Exon 1 and exon 2 | 91 |
| *AT1G08830* | *CSD1* | TCCATGCAGACCCTGATGAC | CCTGGAGACCAATGATGCC | Exon 5 and E6-E7-jn | 102 |
| *AT2G28190* | *CSD2* | GAGCCTTTGTGGTTCACGAG | CACACCACATGCCAATCTCC | Exon 6 and E7-E8-jn | 101 |
| *AT4G25100* | *FSD1* | CTCCCAATGCTGTGAATCCC | TGGTCTTCGGTTCTGGAAGTC | Exon 4 and E6-E7-jn | 101 |
| *AT3G09220* | *preLAC7* | ACCATTGAGGCACTGACCAA | GTACAAAACTTAACGGTGTCTAGGC | Intron 1 and exon 2 | 96 |
| *AT1G08830* | *preCSD1* | ACTGTTGGAGATGATGGTATGCCT | GAGAGAGTAGCGAAATTTGATGCAA | E3-I4-jn and intron 4 | 93 |
| *AT2G28190* | *preCSD2* | TCACTATGACTTAGGCTGCGATTG | GATGGTCCGAATTTGCGATTAA | Intron 7 | 102 |
| *AT4G05105* | *pri-miR397a** | GTGCAGCGTTGATGTAATTTCGTTTTG | ACGATCCGCATACCTGTTTAAGTGTTC | - | 125 |
| *AT5G14545* | *pri-miR398b** | AGTAATCAACGGCTGTAATGACGCTAC | TGACCTGAGAACACATGAAAACGAGAG | - | 202 |
| *AT5G14565* | *pri-miR398c** | TCGAAACTCAAACTGTAACAGTCC | ATTTGGTAAATGAATAGAAGCCACGGGCCACG | - | 241 |
| *AT2G47015* | *pri-miR408** | AGAGAGACAGGGAACAAGCAGAGC | AAGAGGCAGTGCATGGGTAGAGAC | - | 87 |
| *AT4G13554* | *pri-miR857** | TTAACGTCGAGCATTTGAATTTCTAG | ACACCTTCAACATACAAAATAACTTAGAAAA | - | 66 |
