# Supplementary material for: Toxicity responses of Cu and Cd: the involvement of miRNAs and the transcription factor SPL7
Source: BMC Plant Biol. 2016 Jun 28;16:145. doi: 10.1186/s12870-016-0830-4 (PMC4924269; doi:10.1186/s12870-016-0830-4)
Supplement: Additional file 5: — Quantitative real-time PCR parameters according to the Minimum Information for publication of Quantitative real-time PCR Experiments (MIQE) guidelines derived from Bustin et al. [51]. (DOCX 16 kb) [file 12870_2016_830_MOESM5_ESM.docx]

**Additional file 5.** **Quantitative real-time PCR parameters according to the Minimum Information for publication of Quantitative real-time PCR Experiments (MIQE) guidelines derived from Bustin *et al*., 2009.**

**Sample/Template**

Source *Arabidopsis thaliana* roots or leaves (entire rosette) in a hydroponic culture

Method of preservation liquid N_2_

Storage time maximum two weeks at -70°C

Handling frozen

Extraction method columns: RNAqueous Kit* (Life Technologies, Carlsbad, CA, USA)

RNA: DNA-free TURBO DNA-free Kit* (Life Technologies)

Design of intron-spanning primers whenever possible

verification of single peak on dissociation curves

Concentration NanoDrop^®^: ND-1000 Spectrofotometer (ThermoScientific, Wilmington, DE, USA)

**Assay optimisation/validation**

Accession number Supplementary Table 2

Amplicon details exon location and amplicon size: Supplementary Table 2

Primer sequence Supplementary Table 2

*In silico* Primers were blasted using the BLAST tool at <http://arabidopsis.org/>

Empirical Primer concentration of 300 nM, unless stated otherwise

Annealing temperature: 60°C

Priming conditions Combination of oligodT-primers and random hexamers

Linear dynamic range samples are within the range of the efficiency curve

**RT and qPCR**

Protocols SuperScriptTM III Reverse Transcriptase* (Life Technologies)

High-Capacity cDNA Reverse Transcription Kit* (Life Technologies)

Fast SYBR Green* (Life Technologies)

As stated in the Materials and Methods section

Reagents As stated in the Materials and Methods section

No template contol C_q_ and dissociation curve verification

**Data analysis**

Specialist software 7900HT Sequence Detection Software, version 2.3 (Life Technologies)

Statistical justification As stated in the Materials and Methods section and in Table legends

Normalisaton Minimum 3 reference genes selected using GrayNorm (Remans *et al*., 2014)

As stated in the Materials and Methods section

*All procedures were performed according to the manufacturer’s protocol.
